# Prognosis and Dissection of Immunosuppressive Microenvironment in Breast Cancer Based on Fatty Acid Metabolism-Related Signature

**DOI:** 10.3389/fimmu.2022.843515

**Published:** 2022-03-31

**Authors:** Yuhui Tang, Wenwen Tian, Jindong Xie, Yutian Zou, Zehao Wang, Ning Li, Yan Zeng, Linyu Wu, Yue Zhang, Song Wu, Xiaoming Xie, Lu Yang

**Affiliations:** ^1^Department of Breast Oncology, Sun Yat-Sen University Cancer Center, State Key Laboratory of Oncology in South China, Collaborative Innovation Center for Cancer Medicine, Guangzhou, China; ^2^ Department of Radiotherapy, Cancer Center, Guangdong Provincial People’s Hospital, Guangzhou, China

**Keywords:** fatty acid metabolism, breast cancer, prognostic signature, tumor immune microenvironment, immunotherapy, therapy resistance

## Abstract

Fatty acid metabolism has been deciphered to augment tumorigenesis and disease progression in addition to therapy resistance *via* strengthened lipid synthesis, storage, and catabolism. Breast cancer is strongly associated with the biological function of fatty acid metabolism owing to the abundant presence of adipocytes in breast tissue. It has been unraveled that tumor cells exhibit considerable plasticity based on fatty acid metabolism, responding to extra-tumoral and a range of metabolic signals, in which tumor microenvironment plays a pivotal role. However, the prognostic significance of fatty acid metabolism in breast cancer remains to be further investigated. Alongside these insights, we retrieved 269 reliable fatty acid metabolism-related genes (FMGs) and identified the landscape of copy number variations and expression level among those genes. Additionally, 11 overall survival-related FMGs were clarified by univariate Cox hazards regression analysis in The Cancer Genome Atlas (TCGA) and the Molecular Taxonomy of Breast Cancer International Consortium (METABRIC) databases. Subsequently, a prognostic signature based on 6 overall survival (OS)-related FMGs was generated using Lasso Cox hazards regression analysis in TCGA dataset and was validated in two external cohorts. The correlation between the signature and several essential clinical parameters, including T, N, and PAM50 subtypes, was unveiled by comparing the accumulating signature value in various degrees. Furthermore, an optimal nomogram incorporating the signature, age, and American Joint Committee on Cancer (AJCC) stage was constructed, and the discrimination was verified by C-index, the calibration curve, and the decision curve analysis. The underlying implications for immune checkpoints inhibitors, the landscape of tumor immune microenvironment, and the predictive significance in therapy resistance to diverse strategies were depicted ultimately. In conclusion, our findings indicate the potential prognostic connotation of fatty acid metabolism in breast cancer, supporting novel insights into breast cancer patients’ prognosis and administrating effective immunotherapy.

## Introduction

Breast cancer (BC) has now surpassed lung cancer as the leading cause of global cancer incidence in 2020 while as the fifth predominant cause of cancer mortality worldwide (1). Although a host of patients with BC could have an improved prognosis as compared to other solid tumors with multiple treatments, including radical surgery, chemotherapy, radiation therapy, and target therapy, there remain some BC patients attaining unfavorable outcomes due to different heterogeneity (2). Thus, a need for a novel biomarker to identify such BC patients remains.

Fatty acid (FA) metabolism, which is essential for energy production and storage, cell membrane proliferation, and the generation of signaling molecules, has received increasing attention for its pivotal role in cancers (3). In detail, dysregulated *de novo* lipid biosynthesis and exogenous FA uptake could assist cancer cells to not only sustain their rapid proliferative rate but also offer a vital energy source during conditions of metabolic stress (4). Previous studies have delineated that the FA composition of FAs, including the ratios of saturated, monounsaturated, and polyunsaturated FAs, confers significant effects on promoting cell survival for eliminating lipotoxicity and ferroptosis (5). In this context, lots of efforts have been made to develop effective drugs to target FA synthesis for patients with cancer, including BC (6).

Recently, increasing studies have confirmed that the tumor microenvironment (TME) plays a critical role in the development and progression of cancer (7, 8). And metabolic disorders, especially changes in FA metabolism in the microenvironment, have a crucial impact on cancer (9). Moreover, a plethora of studies have demonstrated that tumor-infiltrating immune cells, as an essential component of TME, exert cardinal effects in promoting tumor progression (10, 11). Among these tumor-infiltrating immune cells, regulatory T cells (Tregs) contribute to tumor proliferation and development by suppressing antitumor responses, which induced angiogenesis and metastasis (12). Notably, BC cells have been detected to release free FAs to block the antitumor activity of cytotoxic T cells (13). Stromal cells in the TME, such as tumor-associated adipocytes and cancer-associated fibroblasts (CAFs), could exacerbate cancer–stroma interactions during cancer progression, in which FAs were secreted into the TME that subsequently affected infiltrating immune cell function (14, 15). In the past decade, clinical immunotherapy of BC has achieved great advances with the advent of immune checkpoint inhibitors, including inhibitors targeting programmed cell death-1 (PD-1), programmed cell death ligand-1 (PD-L1), and cytotoxic T-lymphocyte antigen-4 (CTLA-4) (16). Despite this, comprehensive studies of the relationship between FA metabolism and tumor immune microenvironment (TME) were still limited.

Vitally, changes in FA metabolism of tumor cells have been detected to be linked with therapy resistance as well (5). Chemotherapeutic resistance of BC cells has been identified to be correlated to the altered lipid composition of cellular membranes, and chemoresistant cancer cells obtain reduced fluidity of lipid bilayers in the cell membranes, which has disrupted the uptake of chemotherapeutic agents *via* passive diffusion or endocytosis (17, 18). As overexpression of FA synthase (FASN) in BC conferred chemoresistance *in vitro*, pharmacological targeting of FASN could make a range of cancer cell types sensitive to chemotherapy (19–21). Regarding radiotherapy, radioresistant nasopharyngeal cancer (NPG) was reported to acquire an augmented FA oxidation (FAO) rate, which was similar in lung carcinoma cells (22, 23). In addition, on the basis that sex hormones have been confirmed to influence lipid metabolism in hormone­dependent BC, studies on combining lipid­altering agents and endocrine drugs have shown promising efficacy *in vitro* of BC (24). Thus, it remains necessary to further investigate the association of FA metabolism-related genes (FMGs) and therapy resistance in BC, which can impart novel implications into the research in therapy resistance.

In the present study, a reliable signature was established rooted in FMGs, and its prognostic utility was systematically evaluated in BC patients. Additionally, the underlying connotations between the signature and the landscape of TME, namely, the expression level of immune checkpoints, predictive enrichment of tumor-infiltrating immune cells, and expression level of immunosuppressive makers, were unraveled, which offered novel insights for personalized immunotherapy. Ultimately, the prognostic availability of the signature in BC patients subjected to various therapy strategies was validated.

## Materials and Methods

### Data Acquisition and Collection of Fatty Acid Metabolism-Associated Genes

The transcriptome expression data together with detailed clinicopathologic information were acquired from The Cancer Genome Atlas (TCGA) database^1^ (113 normal breast samples and 1,109 BC samples), the Molecular Taxonomy of Breast Cancer International Consortium (METABRIC) database^2^ (1,904 BC samples), and GSE96058 in the Gene Expression Omnibus (GEO) database^3^ (3,409 BC samples). After excluding patients without overall survival (OS) information, we obtained 1,082 patients with breast cancer of TCGA as a training set and 1,903 patients of METABRIC with 3,409 patients of GSE96058 as validation sets for further investigation. Three gene sets concerning FA metabolism, including *KEGG fatty acid metabolism pathways*, *Hallmark fatty acid metabolism genes*, and *Reactome fatty acid metabolism genes*, were obtained from the Molecular Signature Database v7.4 (MSigDB)^4^, and 309 FMGs were collected after the overlapping genes in the above three gene sets were removed (25). Subsequently, 269 reliable FMGs were retrieved for further analysis after employing the above 309 FMGs to intersect with total genes included in TCGA-BRCA, METABRIC, and GSE96058 datasets (**Supplementary Figure 1**).

### Identification of Copy Number Variation Frequency and Differentially Expressed Genes Among Fatty Acid Metabolism-Related Genes

The information on the copy number variation (CNV) of BC was attained from TCGA-BRCA in the UCSC Xena database^5^. Then, the CNV frequency of 269 FMGs was calculated, and the most notable results were visualized with a bidirectional column chart. To identify the differentially expressed genes (DEGs) within FMGs between normal breast samples and BC samples, the “edgR” R package (26) was utilized in the TCGA-BRCA cohort with the significance criteria set to |log2FC| > 1 and false discovery rate (FDR) < 0.05. A volcano plot and a heatmap of these remarkable DEGs were displayed.

### Generation of Overall Survival-Related Fatty Acid Metabolism-Related Genes

TCGA-BRCA (n = 1,082) and METABRIC (n = 1,903) databases were applied to illustrate the potential prognostic significance of 269 FMGs in the BC patients. OS-related FMGs with *p* < 0.05 were generated using univariate Cox hazards regression analysis in two datasets. The overlapping OS-related FMGs were extracted to the next prognostic model construction. Afterward, the “RCircos” R package (27) was used to visualize the expression levels and the location of those genes on chromosomes, and the correlation matrix plot was performed to unravel the correlation features among those genes.

### Construction and Validation of Fatty Acid Metabolism-Relevant Prognostic Signature for Breast Cancer Patients

For the eligible OS-related FMGs, the least absolute shrinkage and selection operator (Lasso) Cox regression analysis was carried out in the training set to construct the statistical prognostic signature. By the means of “glmnet” R package (28), 6 optimal FMGs, expression levels between normal breast tissues and BC tissues along with Kaplan-Meier (K-M) analyses in TCGA-BRCA of which were exhibited respectively, were screened out to establish the best prognostic model. According to the predictive model, the FA metabolism index (FMI) could be exported for each BC patient using the following formula:


FMI=Σ Ei∗βi (Ei represents the expression level of each FMG, and βi represents the corresponding regression coefficient).

To make data and plots more intuitionistic, a linear transformation was performed to adjust the FMI, using the minimum in the total FMI in every single dataset subtracted from the calculated FMI of each patient initially and then dividing the above result by the maximum, which enabled these FMI values to map to the range of 0 to 1. In addition, compared with the median cutoff of FMI, the patients could be divided into high- and low-FMI groups. To evaluate the feasibility of the model, K-M analysis of OS was implemented between the high- and low-FMI groups in three datasets separately. Moreover, disease-free survival (DFS), disease-specific survival (DSS), and progression-free survival (PFS) using K-M analyses were manifested between the high- and low-FMI groups in the training set.

### Comprehensive Assessment of Fatty Acid Metabolism Index and Clinical Parameters in Patients With Breast Cancer

To formulate the availability of FMI applied in clinical situations further, boxplots with Kruskal’s test were depicted to compare the distribution of adjusted FMI value in various degrees of diverse clinicopathologic parameters available in the three datasets. Besides, heatmaps were shown to demonstrate the relevance between every model-contained FMG’s expression level and several clinical indicators, comprising FMI, T, N, American Joint Committee on Cancer (AJCC) stage, and survival status in the training set in addition with FMI, tumor size, positive nodes, PAM50 subtypes, AJCC stage, and survival status in METABRIC dataset.

### Development and Evaluation of Fatty Acid Metabolism-Correlated Clinicopathologic Nomogram

Furthermore, univariate and multivariate Cox regression analyses were conducted to clarify whether the FMI was an independent prognostic predictor of BC. Based on the results above, a FA metabolism-related clinicopathologic nomogram that incorporated FMI to another two clinical characteristics containing age and AJCC stage in the training set was developed using the “rms” and “regplot” R package (29). To confirm the satisfactory predictive discrimination of the nomogram, the calibration curve (30) and the decision curve analysis (DCA) were portrayed for BC patients.

### Conducting Function Enrichment Analysis Within Two Fatty Acid Metabolism Index Groups

Gene Set Enrichment Analysis (GSEA) (31) was conducted to decipher the primarily enriched signaling pathways and biological functions between the high- and low-FMI groups in the training set. “h.hallmark.v7.4.symbols.gmt” [Hallmarks] and “c2.cp.kegg.v7.4.symbols.gmt” [KEGG] were selected as the reference molecular signature databases, and |NES| > 1.5 and FDR *q*-value < 0.1 were considered statistically significant.

### Potential Implications for Immunotherapy Based on the Signature and Tumor-Immune Microenvironment Landscape Estimation

Recent years have witnessed an increasing improvement in immunotherapy and innovative targeted therapy for BC patients. Therefore, we predict the potential influence of immunotherapy according to FMI here by comparing the expression level of several immunologic checkpoints (32) between high- and low-FMI subgroups using the Wilcoxon test. These candidate checkpoints encompassed PDCD1 (PD-1), CD274 (PD-L1), CTLA4, IDO1, CD96, TIGIT, LAG3, and PVR.

The CIBERSORT deconvolution algorithm (33) was utilized to calculate the abundance of 22 tumor immune-infiltrating cell types within the gene expression matrix of TGCA-BRCA cohort in the TME of the high- and low-FMI BC samples. A violin plot was depicted to unveil the results of CIBERSORT analysis while *p* < 0.05 from the Wilcoxon test was considered statistically significant.

To clarify the correlation between FMI signature and TME immunosuppressive factors in BC, several pivotal TME immunosuppressive genes were selected to contrast the expression level in the high-FMI group with that in the low-FMI group, which consisted of IL10, TGF-β, FOXP3, IL6, and FAP with boxplots.

### Prognostic Investigation of Fatty Acid Metabolism Index in Breast Cancer Patients Undergoing Different Treatments

To assess the reliability of prognostic effect of FMI signature for BC patients undergoing different treatment regimens further, K-M analyses of OS were executed between high- and low-FMI subgroups of BC patients receiving chemotherapy, endocrinotherapy, and radiotherapy in the METABRIC and TCGA-BRCA datasets, respectively.

### Cell Lines and Cell Culture

Human BC cell lines were purchased from the American Type Culture Collection. All cell lines were cultured following standard guidelines. All cell lines were maintained without antibiotics in an atmosphere of 5% CO_2_ and 99% relative humidity at 37°C. Cell lines were passaged for fewer than 6 months and were authenticated by short tandem repeat analysis. No mycoplasma infection was found for all cell lines.

### RNA Isolation and Quantitative Real-Time PCR Analysis

Total RNA of cells was extracted with RNA-Quick Purification Kit (ES-RN001, Shanghai Yishan Biotechnology Co., Shanghai, China). The primer sequences are shown in **Supplementary Table S1**. RNA levels were determined by quantitative real-time PCR (qRT-PCR) in triplicate on a Bio-Rad CFX96 using the SYBR Green method (RR420A, Takara, Mountain View, CA, USA). The qRT-PCR plate was employed from NEST (402301, Wuxi NEST Biotechnology Co., Jiangsu, China). The RNA levels were normalized against β-actin RNA using the comparative Ct method.

### Statistical Analysis

All statistical analyses were accomplished *via* R software (Version 4.0.2, http://www.R-project.org). The comparison of each K-M curve contained in this study was completed by the log-rank test. The differences of the expression level of signature-included FMGs in normal and BC tissues, checkpoints, and TME immunosuppressive factors in the low- and high-FMI groups were detected by the Wilcoxon test. And the discrepancies of adjusted FMI value in various clinicopathologic parameters were observed by the Kruskal test. Univariate and multivariate Cox regression analyses were exploited to screen out the OS-related FMGs and the independent prognostic indicators of OS. The correlation matrix plots were displayed by virtue of Spearman’s correlation test. Statistical significance was determined as *p*-value <0.05, and all *p*-values were bilateral.

## Results

### Identification of Prognostic Fatty Acid Metabolism-Related Genes in Breast Cancer Patients

**Figure 1:**
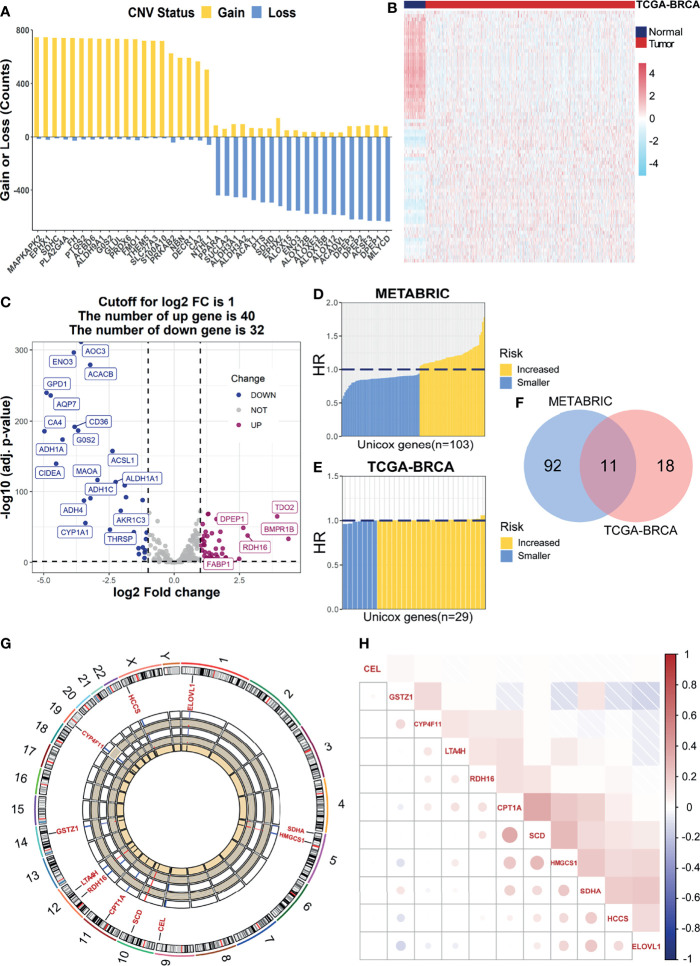
Identification of prognostic FMGs in BC patients. **(A)** The CNV frequency of FMGs in TCGA-BRCA cohort. **(B)** Heatmap of 72 differentially expressed genes from FMGs. **(C)** Volcano plot exhibiting 72 DEGs among FMGs. **(D)** One hundred three prognostic FMGs in METABRIC dataset. **(E)** Twenty-nine prognostic FMGs in TCGA-BRCA dataset. **(F)** Venn diagram to identify 11 overlapping prognostic FMGs. **(G)** The circos plot depicting the location on chromosomes and expression level of 11 prognostic FMGs. **(H)** The correlation matrix plot displaying the correlation features among 11 prognostic FMGs in TCGA-BRCA. FMGs, fatty acid metabolism-related genes; BC, breast cancer; CNV, copy number variation; DEGs, differentially expressed genes; TCGA, The Cancer Genome Atlas.

Initially, we encapsulated the incidence of CNV and identification of DEGs among 269 FMGs in TCGA-BRCA cohort. By investigating the frequency of CNV, we noticed that FMGs have existing prevalent CNV alterations, and the top 20 genes in amplified or deleted CNV status are displayed (**Figure 1A**). Additionally, we detected 72 DEGs by comparing 1,082 BC samples with 113 normal breast samples using the threshold of |log2FC| > 1 and FDR < 0.05. Among the DEGs, there are 40 significantly augmented FMGs in BC patients, while there are 32 FMGs essentially attenuated in BC samples (**Figures 1B, C****)**. To identify the potential prognostic value of each available FMG, a univariate Cox hazards regression analysis was employed to screen out OS-related FMGs in METABRIC and TCGA-BRCA datasets (**Figures 1D, E****)**, which retrieved 103 and 29 significant OS-related FMGs, respectively. Intersecting the results of two cohorts, 11 overlapping OS-related FMGs (HCCS, CPT1A, CYP4F11, HMGCS1, SCD, ELOVL1, GSTZ1, CEL, RDH16, LTA4H, and SDHA) were eligible for further analysis (**Figure 1F**). Moreover, the location on chromosomes and expression level in two datasets of above 11 FMGs were illustrated by a circos plot (**Figure 1G**). And the correlation features among those genes were unraveled *via* a correlation matrix plot (**Figure 1H**).

### Construction of Fatty Acid Metabolism-Relevant Prognostic Signature for Breast Cancer Patients

**Figure 2:**
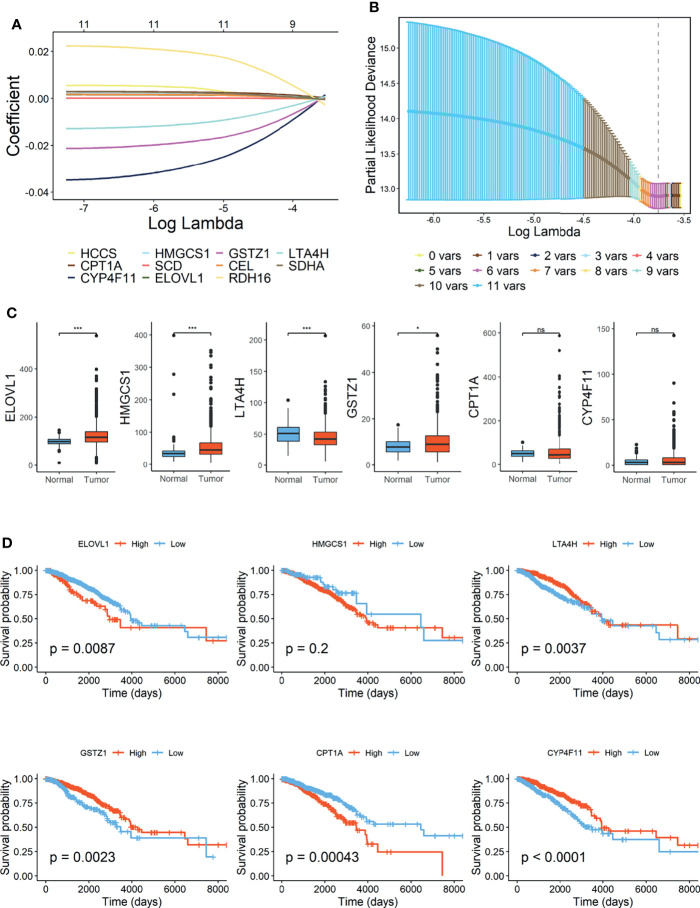
Construction of fatty acid metabolism-related signature in TCGA-BRCA cohort. **(A)** Lasso Cox regression analysis. **(B)** Partial likelihood deviance for the Lasso regression. **(C)** Expression level of 6 FMGs contained in signature. **(D)** Kaplan–Meier (K-M) analyses of OS based on expression level of 6 FMGs. TCGA, The Cancer Genome Atlas; FMGs, fatty acid metabolism-related genes; OS, overall survival. *Means p < 0.05; *** means p < 0.001; ns means no significance.

Lasso Cox regression was performed in TCGA-BRCA cohort with 11 candidate OS-related FMGs aiming to unearth the optimal FMGs for establishing the prognostic signature. Ultimately, six pivotal FMGs were extracted to construct the signature, which embodied CPT1A, CYP4F11, HMGCS1, ELOVL1, GSTZ1, and LTA4H (**Figures 2A, B****)**. Besides, to further dig out the landscape of expression level and independent prognostic capability of each signature-contained FMG, boxplots (**Figure 2C**) of expression level and K-M curves of OS (**Figure 2D**) in the training set were exhibited. From the results, we perceived that the expression levels of HMGCS1, ELOVL1, and GSTZ1 were considerably promoted, while that of LTA4H was relatively diminished in BC samples. However, CPT1A and CYP4F11 were neither increased nor declined substantially. In the separated K-M analyses of OS, the high-expression group of ELOVL1 and CPT1A along with the low-expression group of LTA4H, CYP4F11, and GSTZ1 signified more impaired OS than another comparative group, while the expression level of HMGCS1 exerted a negligible effect on the OS of BC patients. Moreover, the RNA expression levels of ELOVL1 and LTA4H were validated in human BC cell lines (**Supplementary Figure 2**), the result of which illustrated that ELOVL1 was significantly promoted in BC cell lines including BT549, MDA-MB-231, SK-BR-3, and T47D, while LTA4H dwindled in BC cell lines except for SK-BR-3 compared with breast epithelial cell line MCF10A.

**Figure 3:**
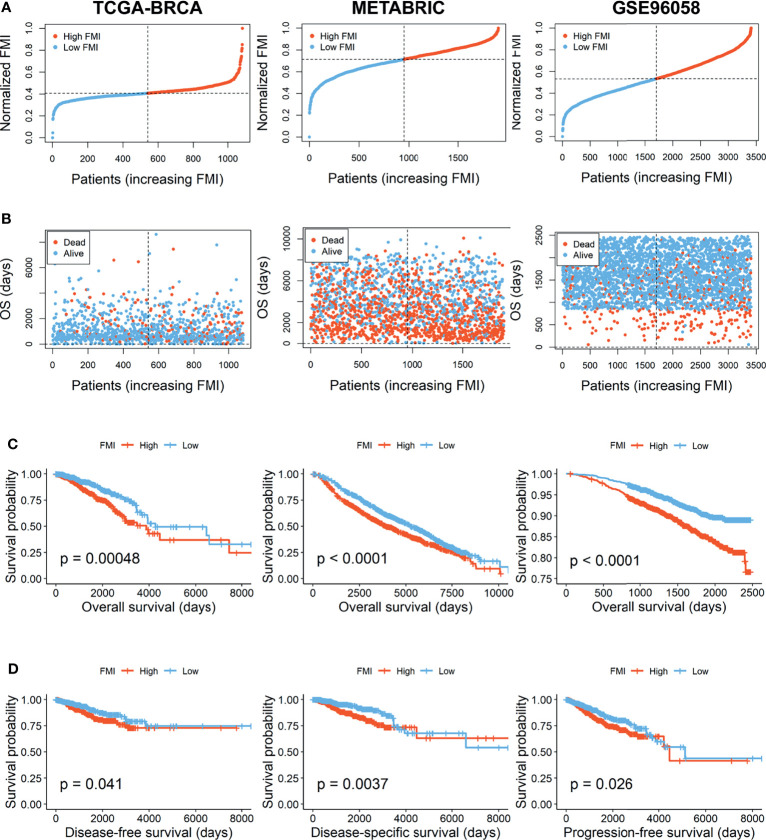
Evaluation and validation of the utility of FMI in training set and validation sets. **(A)** Distribution of the patients’ normalized FMI score. **(B)** Patients’ overall survival time along with their FMI score. **(C)** K-M analyses of OS between high- and low-FMI groups. **(D)** K-M analyses of DFS, DSS, and PFS between high- and low-FMI groups in training set. FMI, fatty acid metabolism index; K-M, Kaplan–Meier; OS, overall survival; DFS, disease-free survival; DSS, disease-specific survival; PFS, progression-free survival.

According to the signature, the FMI of each patient was calculated as follows: FMI = Expression of CPT1A * 0.000952 − Expression of CYP4F11 * 0.003123 + Expression of HMGCS1 * 0.000327 + Expression of ELOVL1 * 0.000245 − Expression of GSTZ1* 0.002498 − Expression of LTA4H * 0.000609. Furthermore, the BC patients could be separated into the high- and low-FMI groups based on the median value of FMI in the training set, and FMI was adjusted to make the data more straightforward (**Figure 3A**). The proportion of dead patients in the high-FMI group was larger than that in the low-FMI group in TCGA-BRCA dataset (**Figure 3B**). To evaluate the prognostic feasibility of FMI, K-M analyses were conducted to decipher that the high-FMI group had vitally worse OS than the low-FMI group (**Figure 3C**), as the same in the assessment of DSS, DFS, and PFS in the training set (**Figure 3D**).

### Validation of Signature Based on 6 Fatty Acid Metabolism-Related Genes

To further verify the prognostic significance of FMI, which was established based on 6 FMGs in the training set, the same investigations were implemented in the two external validation sets, METABRIC and GSE96058. With the same calculation formula of FMI, BC patients were segregated into the high- and low-FMI groups in the two datasets (**Figure 3A**). As expected, the number of BC patients with dead status was gradually promoted with the increase of FMI in GSE96058, but not in METABRIC (**Figure 3B**). Consistent with the training set, the high-FMI group implicated more deteriorated OS than the low-FMI group in validation sets (**Figure 3C**).

### Integrated Assessment of Fatty Acid Metabolism Index and Clinical Parameters in Patients With Breast Cancer

**Figure 4:**
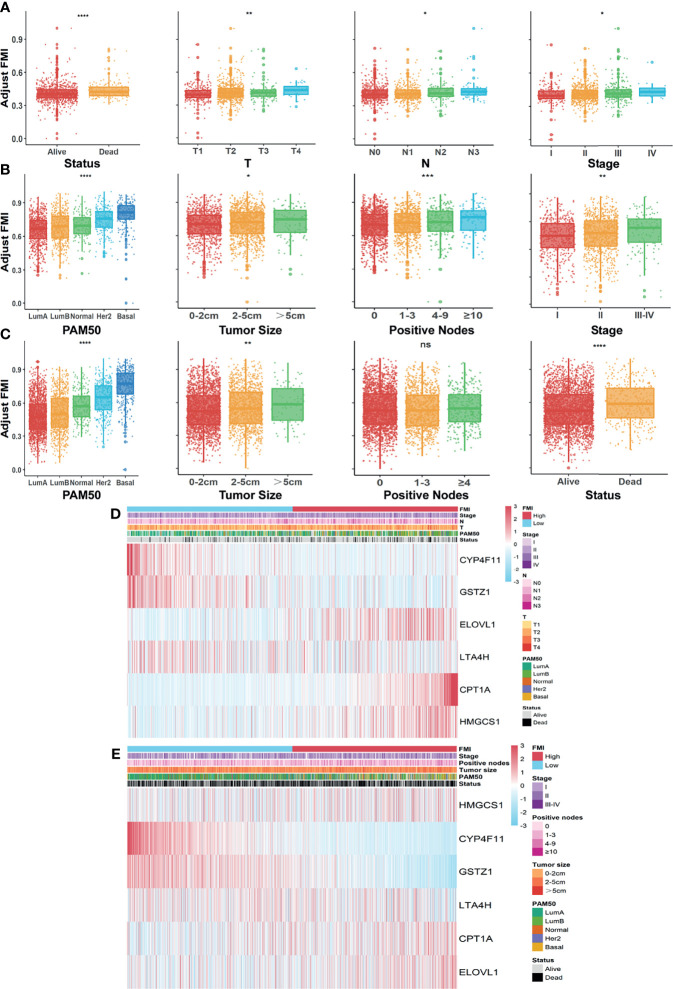
Systematic assessment of FMI and clinical parameters in BC patients. Comparison of adjusted FMI in various degrees of diverse clinical parameters in TGCA-BRCA **(A)**, METABRIC **(B)**, and GSE96058 **(C)** datasets. Heatmaps integrating FMI and clinical parameters in training set **(D)** and in METABRIC cohort **(E)**. FMI, fatty acid metabolism index; BC, breast cancer; TCGA, The Cancer Genome Atlas; METABRIC, Molecular Taxonomy of Breast Cancer International Consortium. *Means p < 0.05; ** means p < 0.01; *** means p < 0.001; **** means p < 0.0001; ns means no significance.

To formulate the availability of FMI in predicting other clinical parameters, we further ascertained the relationship between the FMI and the clinical characteristics. In the training set, there were prominent discrepancies of adjusting FMI in various degrees of diverse clinical parameters including survival status, clinicopathologic T, clinicopathologic N, and AJCC stage (all *p* < 0.05), and the higher FMI was likely associated with the severer degree of the above clinical features in tendency (**Figure 4A**). Likewise, conspicuous differences were reconfirmed in distinctive levels of diversified clinical parameters, containing PAM50 subtypes, tumor size, positive nodes, and AJCC stage in the METABRIC validation set (**Figure 4B**) together with PAM50 subtypes, tumor size, and patients’ status, except for positive nodes in the GSE96058 dataset (**Figure 4C**), which hinted that the more elevated FMI was connected to the clinical factors, implying the more impaired survival of BC patients. The integrated correlation analysis between the expression level of each FMI-involved FMG and the clinical parameters available in TCGA-BRCA and METABRIC datasets are delineated in **Figures 4D, E**.

### Development and Evaluation of Fatty Acid Metabolism-Correlated Clinicopathologic Nomogram

**Figure 5:**
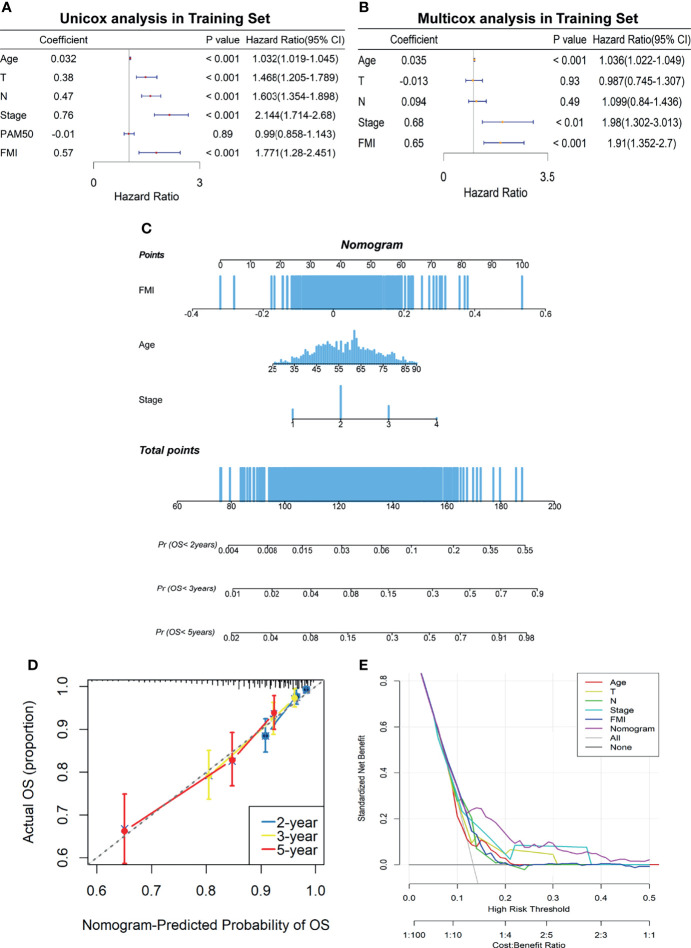
Establishment of FA metabolism-related clinicopathologic nomogram. **(A)** Univariate Cox regression analysis of the signature FMI and clinical parameters. **(B)** Multivariate Cox regression analysis of the signature FMI and clinical parameters. **(C)** Development of a prognostic nomogram to predict 2-, 3-, and 5-year OS in BC patients of the training set. **(D)** Calibration curve to evaluate the consistency of predicted and actual OS. **(E)** Decision curve analysis (DCA) to assess the clinical decision-making benefits of the nomogram. FA, fatty acid; FMI, fatty acid metabolism index; OS, overall survival; BC, breast cancer.

To elucidate whether FMI was an independent prognostic indicator of BC, univariate and multivariate Cox regression analyses were carried out in the training set (**Figures 5A, B**), the results of which illuminated that age, T, N, AJCC stage, and FMI were remarkably linked to OS of BC patients in the univariate Cox analysis (all *p* < 0.001) while only age, AJCC stage, and FMI were independent prognostic predictors in the multivariate Cox analysis (all *p* < 0.01). Based on the results above, a clinicopathologic nomogram with an optimal concordance index (C-index, 0.76) that incorporated FMI to another two clinical characteristics containing age and AJCC stage was developed to predict individual OS of 2, 3, and 5 years (**Figure 5C**). To confirm the satisfactory predictive discrimination of the nomogram, the calibration plot was portrayed and discovered to be closer to the ideal curve (**Figure 5D**), which presented the perfect stability of the nomogram. Subsequently, DCA elicited that the nomogram attained a greater net benefit than the single independent clinical feature (**Figure 5E**). In a nutshell, the efficiency of the prognostic nomogram was clarified from multiple aspects.

### Gene Set Enrichment Analysis Between High and Low Fatty Acid Metabolism Index Groups

**Figure 6:**
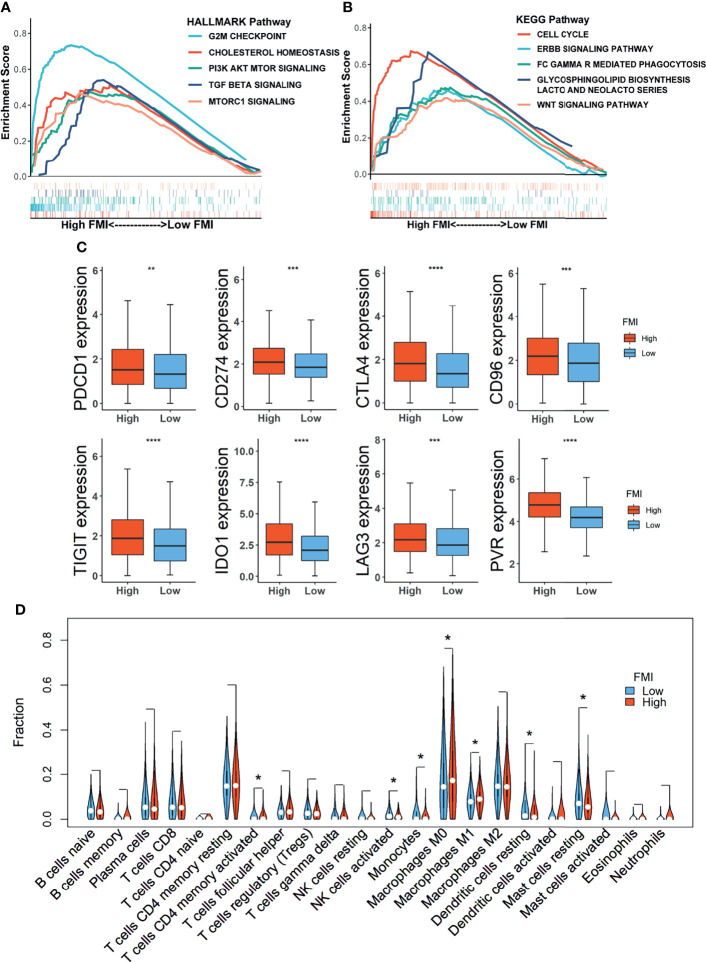
Gene Set Enrichment Analysis (GSEA) and landscape of tumor immune microenvironment between the high- and low-FMI groups. **(A, B)** Results of GSEA in TCGA-BRCA cohort. **(C)** Expression level of immune checkpoints in training set. **(D)** Violin plots of the proportions of 22 immune-infiltrating cells in total BC patients. FMI, fatty acid metabolism index; TCGA, The Cancer Genome Atlas; BC, breast cancer. *Means p < 0.05; **means p < 0.01; ***means p < 0.001; ****means p < 0.0001.

GSEA was conducted to decipher the primarily enriched signaling pathways and biological functions between the high- and low-FMI groups in the training set. As shown in **Figures 6A, B**, the results using the Hallmark database demonstrated that G2/M checkpoint, cholesterol homeostasis, PI3K–AKT–mTOR signaling, TGF beta signaling, and mTORC1 signaling were fundamentally enriched in the high-FMI group, while cell cycle, ERBB signaling pathway, FC Gamma R-mediated phagocytosis, glycosphingolipid biosynthesis lacto and neolacto series, and WNT signaling pathway were abundant in the high-FMI group through the GSEA results using KEGG database.

### Potential Implications for Immunotherapy and Landscape of Tumor-Immune Microenvironment

Emerging novel molecules for immunotherapy and targeted therapy, such as immune checkpoints, were currently observed and assessed in preclinical or clinical trials for the treatment of BC patients. Therefore, the comparison of expression levels of 8 candidate immune checkpoints between the low-FMI and high-FMI groups in the training set unveiled that expression levels of those targets encompassing PDCD1 (PD-1), CD274 (PD-L1), CTLA4, IDO1, CD96, TIGIT, LAG3, and PVR were all significantly magnified in the high-FMI groups (**Figure 6C**). Similar results also existed in another two validation sets (**Supplementary Figure 3**). These results shed light on that BC patients with higher FMI might obtain a more enhanced response to therapies targeting the checkpoints above.

To further reveal the landscape of TME, an analysis of immune-infiltrating cells in the TME of BC as defined by FMI in the training set was accomplished, the result of which uncovered that activated memory CD4+ T cells and macrophages M0 and M1 were notably strengthened in the TME of the high-FMI group, while activated natural killer (NK) cells, monocytes, resting dendritic cells (DCs), and resting mast cells were markedly activated in the low-FMI group (**Figure 6D**). Then, we also dissected the distribution of LM22 in BC of basal and non-basal subtypes separately (**Supplementary Figure 4**). In the basal subtype, CD8+ T cells were attenuated, while macrophage M0 was intensified significantly in the high-FMI group. In the non-basal subtype, naive B cells, activated NK cells, monocytes, resting DCs, and resting mast cells were enriched, while activated memory CD4+ T cells and macrophages M0 were dampened substantially in the low-FMI group. Interestingly, the predominant difference in the enrichment of infiltrating M1 macrophages was not detected in the basal subtype or non-basal subtype.

**Figure 7:**
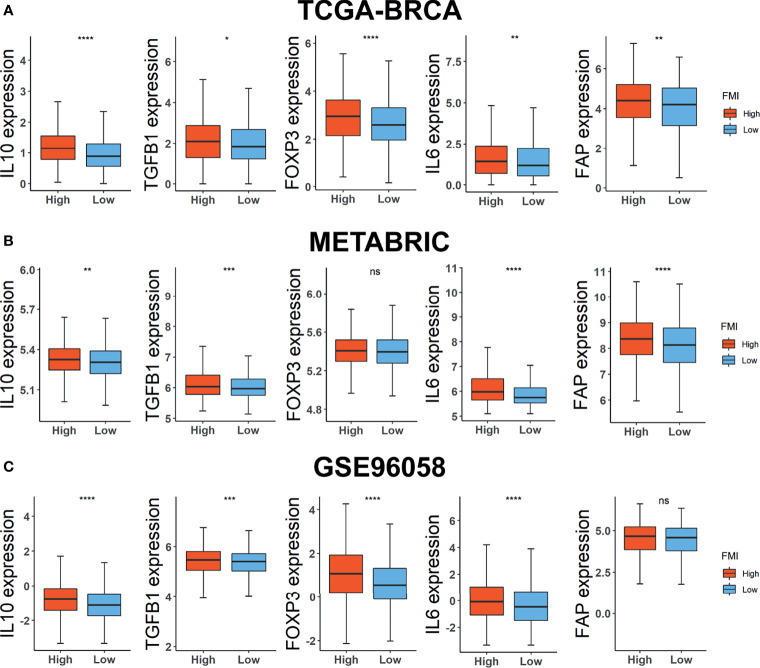
Investigations of TME immunosuppressive cytokines and markers. Expression level of IL10, TGF-β, FOXP3, IL6, and FAP in TCGA-BRCA **(A)**, METABRIC **(B)**, and GSE96058 **(C)** datasets. TME, tumor microenvironment; TCGA, The Cancer Genome Atlas; METABRIC, Molecular Taxonomy of Breast Cancer International Consortium. * Means p < 0.05; ** means p < 0.01; *** means p < 0.001; **** means p < 0.0001; ns means no significance.

Consequently, we carried on scrutinizing the correlation between FMI signature and the expression level of TME immunosuppressive factors further, which comprises the pivotal cytokines of immunosuppressive TME, IL-10 and TGF-β (34), Treg marker FOXP3 (35), cancer-associated adipocytes activated marker IL-6 (36), and CAF marker FAP (37). In the training set, the expression levels of the five above-mentioned cytokines and markers were intensified in the high-FMI (**Figure 7A**). A similar tendency occurred in the two external validation datasets except for FOXP3 in METABRIC and FAP in GSE96058, the expression levels of which differed slightly in two comparative FMI groups (**Figures 7B, C****)**. Collectively, the results above indicated that a strong immunosuppressive TME, which contributed to the immune escape of tumor cells and the impeded prognosis, might remain in the BC patients of the high-FMI group.

### Prognostic Investigation of Fatty Acid Metabolism Index in Breast Cancer Patients Undergoing Different Treatments

**Figure 8:**
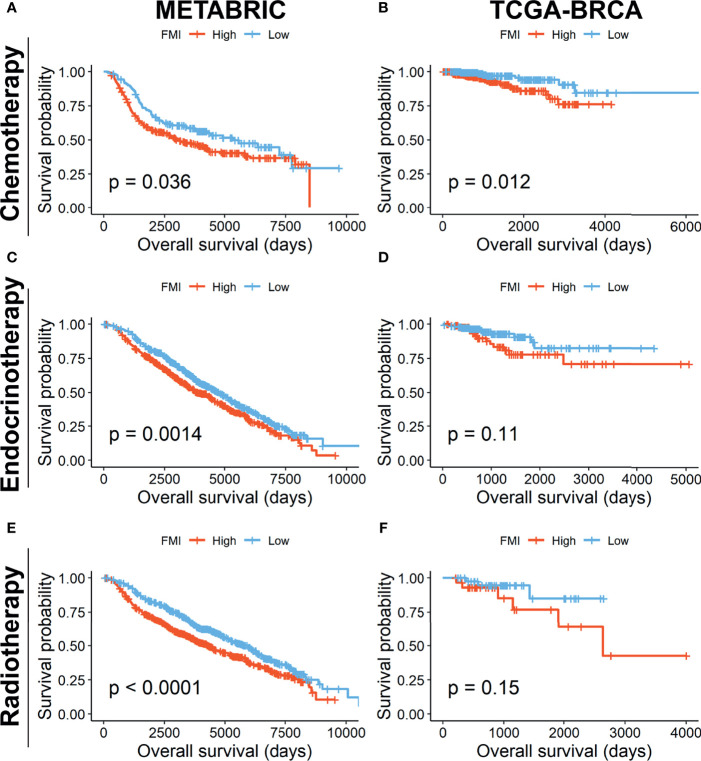
Prognostic validation of FMI in BC patients undergoing different treatment regimens. K-M analyses of OS in BC patients undergoing chemotherapy **(A, B)**, endocrinotherapy **(C, D)**, and radiotherapy **(E, F)** between high- and low-FMI groups in METABRIC and TCGA-BRCA cohorts, respectively. FMI, fatty acid metabolism index; BC, breast cancer; K-M, Kaplan–Meier; OS, overall survival; METABRIC, Molecular Taxonomy of Breast Cancer International Consortium; TCGA, The Cancer Genome Atlas.

Furthermore, we investigated the reliability of the prognostic effect of FMI signature for BC patients receiving different treatment regimens. In the METABRIC set, the BC patients undergoing traditional chemotherapy (n = 412) in the high-FMI group was detected to gain worse OS than those in the low-FMI group (**Figure 8A**), as is the same situation in the patients subjected to endocrinotherapy (n = 1,174) and radiotherapy (n = 1,137) (**Figures 8B, C****)**. Likewise, in TCGA-BRCA set, the BC patients accepting chemotherapy (n = 499) in the high-FMI group acquired shortened OS time (**Figure 8D**), while there was limited significance in the patients going through endocrinotherapy (n = 264) and radiotherapy (n = 72) between two FMI groups (**Figures 8E, F****)** likely due to the lack of samples.

## Discussion

Accumulating evidence has delineated that dysregulated metabolism in cancer cells and tumor environment is of pivotal contribution to the cancer progression, treatment, recurrence, and metastasis, including FA metabolism (38). FA metabolism, encompassing a broad range of metabolic signaling pathways, influences cancer cell biology notably depending on the synthesis of lipid building blocks for membranes and the energy production and storage (3). Moreover, it has been appreciated that the FA composition of the cell membrane, such as the ratios of saturated, monounsaturated, and polyunsaturated FAs, exerts vital effects on enhancing cell survival while diminishing lipotoxicity and ferroptosis (5). Cancer cells can obtain FAs from exogenous sources or synthesize them endogenously through lipogenic pathways in a dysfunctional way, which subsequently disrupt homeostasis and strengthen cellular stress owing to the excessive accumulation of lipids or a shift in saturated and unsaturated FA levels (39). As a molecularly heterogeneous malignant tumor, BC is confirmed to be strongly associated with the biological functions of FA metabolism given the substantial presence of adipocytes in breast tissue (40). Nonetheless, the essential molecular markers related to FA metabolism in BC still remain dissected thoroughly.

More recently, it has been a prevalent issue to establish the prognostic signature based on genesets correlated to specific biological characteristics in cancer research (41–43). It is well known that the decision-making capability of a clinical doctor to administrate personalized treatment could be improved *via* an accurately predicted prognosis through stratifying the patients into the high- or low-risk group on the basis of a reliable predictive signature. Accordingly, in the current study, we comprehensively and systematically constructed a signature based on the FMGs, further anatomized its actual clinical value, and assessed the landscape of TME for BC patients as defined by an established signature to support essential preclinical implications in the field of FA metabolism in cancer research.

Initially, 269 reliable FMGs were retrieved, and the prevalent CNV alterations and DEGs among them were identified in BC samples, which confirmed the significant effect of FA metabolism in malignant cancerous lesions. Eleven OS-related FMGs were further extracted by univariate Cox hazards regression analysis, and 6 decisive OS-related FMGs were screened out to constitute an optimal prognostic signature using Lasso Cox regression analysis, namely, FMI. And the excellent utility was validated in three cohorts of BC patients to predict not only their OS but also DSS, DFS, and PFS in the high- and low-FMI groups. There have been a host of credible studies on FMI-contained FMGs in multiple cancer types, which could verify the reliability of the signature to some extent. For instance, carnitine palmitoyl transferase 1A (CPT1A), as the rate-limiting enzyme of long-chain FA transport into the mitochondria for FAO, could be enhanced in BC cells when responding to adipocyte lipolysis (44, 45). Besides, HER2-expressing radioresistant BC cells and radioresistant BC stem cells could be characterized by high expression of CPT1A and increased FAO, and patients with high CPT1A have a poor prognosis (46). CYP4F11, one of omega FA metabolizing enzymes, can metabolize the compounds into irreversible inhibitors of stearoyl CoA desaturase (47), and its expression level is predominantly and independently associated with the OS in colorectal cancer (48). Additionally, 3-hydroxy-3-methylglutaryl-coenzyme A synthase 1 (HMGCS1), as one of cholesterol biosynthesis genes, is impeded in expression level when utilizing the selective PI3Kbeta inhibitor AZD8186 in PTEN-null triple-negative breast cell lines and tumor xenografts (49). Elongation of very-long-chain FAs 1 (ELOVL1) is a single elongase catalyzing the synthesis process of both saturated and monounsaturated very-long-chain FAs (VLCFAs) and is detected at high levels in the phospholipids of BC samples, the silencing of which could hamper the viability and lipidomic profiles of BC cells (50). Glutathione transferase zeta 1 (GSTZ1), the enzyme responsible for conversion of dichloroacetate to its inactive metabolite, is observed to decline in liver cancer but increase in BC, the underlying mechanism of which associated with FA metabolism in BC demands further investigations (51). Leukotriene B4-synthesizing enzyme (LTA4H) is involved in reducing breast tumor growth and improving chemotherapy in lipid sphingosine-1-phosphate receptor 4 (S1PR4) ablation manner and *via* CD8+ T-cell expansion (52). Consistent with the above studies, we examined the expression of HMGCS1, ELOVL1, and GSTZ1 and found that they were considerably promoted, while that of LTA4H was relatively curbed in BC samples, in addition with the significant correlation between the expression level of 5 FMGs except for HMGCS1 and OS of BC patients.

Subsequently, the vital association between the FMI signature and clinical parameters accessible in three datasets, including survival status, T or tumor size, N or positive nodes, AJCC stage, and PAM50 subtypes, were clarified, and the results displayed hinted that the more augmented FMI value was connected to the higher degree of clinical characteristics, implying the more impaired survival of BC patients. Furthermore, the FMI signature was ascertained to be an independent prognostic indicator, even when adjusted for other clinical variables through univariate and multivariate Cox regression analyses. Ultimately, a predictive nomogram that incorporated FMI, age, and AJCC stage was developed, and the practicability of the designed nomogram was assessed by C-index, calibration plot, and DCA, which exhibited its satisfactory predictive discrimination in monitoring OS of BC patients.

Moreover, GSEA was conducted between the high- and low-FMI groups; G2/M checkpoint, cholesterol homeostasis, PI3K–AKT–mTOR signaling, TGF beta signaling, and mTORC1 signaling, cell cycle, ERBB signaling pathway, FC Gamma R-mediated phagocytosis, glycosphingolipid biosynthesis lacto and neolacto series, and Wnt signaling pathway were enriched in the high-FMI group. Notably, it has been reported that several metabolic processes are activated by PI3K–AKT–mTOR signaling, comprising oxidative phosphorylation by promoting mitochondrial biosynthesis, sustaining *de novo* nucleotide synthesis, and lipogenesis (3). In the processing of the lipogenesis, the conversion of transcriptional regulator SREBP1 to its mature active station is strongly affected by PI3K–AKT–mTORC1-dependent mechanisms (53). Hence, the expression of vital lipogenic enzymes, like FASN, ACC1, and ACLY, is attenuated following mTORC1 inhibition by acute rapamycin inhibition or Raptor knockdown (54). Taken together, our results have reconfirmed the fundamental role of PI3K–AKT–mTOR signaling in lipid acid metabolism for BC patients.

Currently, a novel strategy of cancer therapy, immunotherapy, is increasingly gaining attention in multiple cancer types, including BC, yet distinguishing more precisely advisable patients for immunotherapy remains to be observed (14). Immune checkpoint inhibitors, as an indispensable strategy in immunotherapy, have been presently employed and assessed in preclinical or clinical trials for the treatment of patients with cancer. Therefore, we implemented the comparison of the expression level of 8 selective immune checkpoints between the high- and low-FMI groups of BC patients to attain the potential implications for immunotherapy. The results unveiled that the expression level of all targeted checkpoints was significantly magnified in high-FMI groups, which indicated that BC patients with higher FMI might obtain a more enhanced response to therapies targeting these checkpoints. Otherwise, immune checkpoint signaling can create an immunosuppressive environment allowing the tumor cell to escape from immune system-mediated destruction, for which the immune cell can be alleviated from eliciting a cytotoxic response upon the continuing activation of checkpoints (55). It is well established that the TME exerts essential effects in the lipid acid metabolic processing between cancer cells and the entire population of immune and stromal cells (38). Consequently, the landscape of TME between the high- and low-FMI groups signified that activated memory CD4+ T cells and macrophages M0 and M1 were notably strengthened for total BC patients in the high-FMI, while activated NK cells, monocytes, resting DCs, and resting mast cells were markedly activated in the low-FMI group. Furthermore, we dissected the abundance of tumor-infiltrating immune cells in basal and non-basal subtypes in a more detailed manner. In the previous study, accumulating concentration of FFAs from circulation or within the TME correlates with reduced CD8+ cytotoxic T lymphocyte activity (13), which is consistent with the result of the basal subtype. Besides, exogenous lipids can disrupt the metabolic programming in the activation of NK cells, showing protective antitumor and long-lasting immunity, and negatively affect NK cell effector functions and their ability to respond to stimuli (56), which is in line with our results. It has been reported that increased lipid accumulation within lipid droplets in tumor-associated DCs causes DC dysfunction by reducing antigen presentation and results in poor stimulation of T-cell responses (57, 58), contributing to the strengthened immunosuppressive TME. As for the effect of M0 macrophages on tumors, they actually were often believed to have an antitumor effect, while there exist some studies investigating their pro-tumor effect and relevance in the inferior survival of patients with cancer. For instance, in Carlos Caldas’ study, it was delineated that M0 macrophages emerged as one of the immune-infiltrating cell subsets most strongly associated with poor outcome of the BC, regardless of estrogen receptor (ER) status (59). Meanwhile, it is reported that a higher fraction of M0 macrophages in ER-positive BC was correlated with worse DFS and, in ER-positive/HER2-negative BC, with inferior OS (60). In a nutshell, the results of our study and the aforementioned studies might indicate that M0 macrophages could exert double-edged effects on breast cancer, which deserves further examination. M1 macrophages are considered to have antitumoral properties due to their pro-inflammatory response to cancer cells (61), which is in contradiction of the LM22 analysis result on total BC. However, the predominant difference of M1 macrophages was not detected in the basal subtype or non-basal subtype, which implicated that the usage of FMI in predicting the abundance of M1 macrophages remains for reconfirmation. Collectively, the result implicated further that the high-FMI group might acquire more immunosuppressive TME than the low-FMI group for BC patients.

Although there is a slight discrepancy in the enrichment of Tregs between two FMI groups, we need to notice the pivotal role of Tregs in immunosuppressive TME with dysregulated FA metabolism. Tregs are a subset of CD4+ T cells that highly express FOXP3, a vital regulator of Treg development and function that promotes FA uptake and FAO and enhances Treg resistance to lipotoxic environments to allow for expansion (62). In addition, the regulatory cytokines, including IL-10 and TGF-β, could be secreted by Tregs to sustain the immunosuppressive microenvironment to assist cancer cells during immune escape (63). Meanwhile, attention should also be paid to two types of stromal cells, tumor-associated adipocytes and CAFs. Once adipocytes are activated by cancer cells, they will ultimately secrete enhanced levels of pro-inflammatory cytokines, including interleukin-6 (IL-6), which are also secreted by cancer cells and contribute to inducing the release of FAs from adipocyte triglyceride stores (36, 64) so that the progression of cancer could be facilitated. Likewise, CAFs, the marker of which is FAP, generate the upregulation of FATP1 in human triple-negative BC cells, contributing to an increase in exogenous FA uptake from the TME (65) and can additionally transfer lipids to cancer cells through ectosomes, which have been illustrated to boost cancer cell proliferation (66). Accordingly, we contrasted the expression level of IL-10, TGF-β, FOXP3, IL-6, and FAP between the high- and low-FMI groups, the outcome of which reconfirmed that strong immunosuppressive TME exists in BC patients with high FMI.

In addition, FA metabolism is detected to be associated with the resistance to a range of cancer treatments, such as chemotherapy, endocrine-targeted therapy, and radiotherapy (5). The altered lipid composition of cellular membranes has long been connected to the response and resistance of tumor cells to chemotherapeutic agents. In detail, a reduced fluidity of lipid bi-layers in the membranes, namely, based on the predominance of saturated fatty acyl-chains in membrane lipids, particularly for lipogenic tumor cells, and increased sphingomyelin or cholesterol content and motivated the chemotherapy resistance in an essential way (17). A commonly reported feature of endocrine therapy-resistant BC cell line models compared to isogenic sensitive lines is sterol regulatory element-binding protein 1 (SREBP)-driven upregulation of genes involved in lipid (notably cholesterol) biosynthesis, and targeting of SREBP was effective in reducing the growth of these resistant cell lines (67, 68). Also, cell lines of NPG and BC that are resistant to radiation therapy commonly feature enhanced rates of FAO along with intensified expression of CPT1A (22). Consequently, the availability of signature in predicting OS of BC patients undergoing different therapy regimens was verified through K-M analyses, which depicted that the BC patients in the high-FMI group had more shortened OS time, regardless if under chemotherapy, endocrinotherapy, or radiotherapy, likely owing to the therapy resistance induced by dysfunctional FA metabolism.

This study carried out a systematic analysis of fatty acid metabolism-related transcriptomic profiling and constructed a prognostic signature FMI on the basis of OS-relevant FMGs in BC patients. Nevertheless, there remain several limitations that should be contemplated when interpreting the results. Although there is significance in predicting the response to immune checkpoint inhibitors, this requires validation in another cohort of BC patients undergoing immunotherapy. Besides, the profound mechanism of signature-contained FMGs in the form of immunosuppressive TME and therapy resistance demands assessments furthermore.

## Conclusion

In conclusion, our study constructed a reliable predictive signature based on FA metabolism-related genes. The signature was clarified as an independent prognostic factor, and a nomogram with high practicability comprising our designed signature was established. The underlying correlation between our signature and the immunosuppressive TME was deciphered. The implications for therapy resistance defined by a signature were unraveled. Briefly, our study supported the pivotal preclinical significance of FA metabolism to cancer research.

## Data Availability Statement

The original contributions presented in the study are included in the article/**Supplementary Material**. Further inquiries can be directed to the corresponding authors.

## Ethics Statement

Consent from all participants was obtained through The Cancer Genome Atlas (TCGA), the Molecular Taxonomy of Breast Cancer International Consortium (METABRIC), and the Gene Expression Omnibus (GEO) databases.

## Author Contributions

All authors participated in the present study, including conception and design LY and XX, data collection YT, WT, JX, YZo, ZW, NL, YZe, LW, YZh, and SW, data analysis YT and JX, drafting the article or critically revision YT, WT and LY, and study supervision LY and XX. All authors have read and approved the final version submitted.

## Funding

This study was supported by the National Natural Science Foundation of China (81872152 to XX) and the National Natural Science Foundation of Guangdong Province, China (2022A1515012536 to LY).

## Conflict of Interest

The authors declare that the research was conducted in the absence of any commercial or financial relationships that could be construed as a potential conflict of interest.

The reviewer MX has declared a shared parent affiliation with the authors YT, WT, JX, YZo, ZW, NL, YZe, LW, YZh, SW, XX to the handling editor at the time of review.

## Publisher’s Note

All claims expressed in this article are solely those of the authors and do not necessarily represent those of their affiliated organizations, or those of the publisher, the editors and the reviewers. Any product that may be evaluated in this article, or claim that may be made by its manufacturer, is not guaranteed or endorsed by the publisher.

## Footnotes

1
https://portal.gdc.cancer.gov/repository.

2
http://www.cbioportal.org/.

3
https://www.ncbi.nlm.nih.gov/geo/.

4
http://www.gsea-msigdb.org/gsea/msigdb/index.jsp.

5
https://xenabrowser.net/datapages/.

## References

[1] Sung H Ferlay J Siegel RL Laversanne M Soerjomataram I Jemal A. Global Cancer Statistics 2020: GLOBOCAN Estimates of Incidence and Mortality Worldwide for 36 Cancers in 185 Countries. CA Cancer J Clin (2021) 71(3):209–49. doi: 10.3322/caac.21660 33538338

[2] Hua X Long ZQ Zhang YL Wen W Guo L Xia W. Prognostic Value of Preoperative Systemic Immune-Inflammation Index in Breast Cancer: A Propensity Score-Matching Study. Front Oncol (2020) 10:580. doi: 10.3389/fonc.2020.00580 32373539 PMC7186330

[3] Koundouros N Poulogiannis G. Reprogramming of Fatty Acid Metabolism in Cancer. Br J Cancer (2020) 122(1):4–22. doi: 10.1038/s41416-019-0650-z 31819192 PMC6964678

[4] Rohrig F Schulze A. The Multifaceted Roles of Fatty Acid Synthesis in Cancer. Nat Rev Cancer (2016) 16(11):732–49. doi: 10.1038/nrc.2016.89 27658529

[5] Hoy AJ Nagarajan SR Butler LM. Tumour Fatty Acid Metabolism in the Context of Therapy Resistance and Obesity. Nat Rev Cancer (2021) 21(12):753–66. doi: 10.1038/s41568-021-00388-4 34417571

[6] Sardesai SD Thomas A Gallagher C Lynce F Ottaviano YL Ballinger TJ. Inhibiting Fatty Acid Synthase With Omeprazole to Improve Efficacy of Neoadjuvant Chemotherapy in Patients With Operable TNBC. Clin Cancer Res (2021) 27(21):5810–7. doi: 10.1158/1078-0432.CCR-21-0493 34400413

[7] Ferguson LP Diaz E Reya T. The Role of the Microenvironment and Immune System in Regulating Stem Cell Fate in Cancer. Trends Cancer (2021) 7(7):624–34. doi: 10.1016/j.trecan.2020.12.014 PMC8318571 33509688

[8] Micalizzi DS Ebright RY Haber DA Maheswaran S. Translational Regulation of Cancer Metastasis. Cancer Res (2021) 81(3):517–24. doi: 10.1158/0008-5472.CAN-20-2720 PMC7854484 33479028

[9] Elia I Haigis MC. Metabolites and the Tumour Microenvironment: From Cellular Mechanisms to Systemic Metabolism. Nat Metab (2021) 3(1):21–32. doi: 10.1038/s42255-020-00317-z 33398194 PMC8097259

[10] Milo I Bedora-Faure M Garcia Z Thibaut R Perie L Shakhar G. The Immune System Profoundly Restricts Intratumor Genetic Heterogeneity. Sci Immunol (2018) 3(29). doi: 10.1126/sciimmunol.aat1435 30470696

[11] Li X Wenes M Romero P Huang SC Fendt SM Ho PC. Navigating Metabolic Pathways to Enhance Antitumour Immunity and Immunotherapy. Nat Rev Clin Oncol (2019) 16(7):425–41. doi: 10.1038/s41571-019-0203-7 30914826

[12] Lan HR Du WL Liu Y Mao CS Jin KT Yang X. Role of Immune Regulatory Cells in Breast Cancer: Foe or Friend? Int Immunopharmacol (2021) 96:107627. doi: 10.1016/j.intimp.2021.107627 33862552

[13] Kleinfeld AM Okada C. Free Fatty Acid Release From Human Breast Cancer Tissue Inhibits Cytotoxic T-Lymphocyte-Mediated Killing. J Lipid Res (2005) 46(9):1983–90. doi: 10.1194/jlr.M500151-JLR200 15961785

[14] Li Z Sun C Qin Z. Metabolic Reprogramming of Cancer-Associated Fibroblasts and Its Effect on Cancer Cell Reprogramming. Theranostics (2021) 11(17):8322–36. doi: 10.7150/thno.62378 PMC8343997 34373744

[15] Man K Kallies A Vasanthakumar A. Resident and Migratory Adipose Immune Cells Control Systemic Metabolism and Thermogenesis. Cell Mol Immunol (2022) 19(3):421–31. doi: 10.1038/s41423-022-00844-7 PMC8891307 34837070

[16] Gaynor N Crown J Collins DM. Immune Checkpoint Inhibitors: Key Trials and an Emerging Role in Breast Cancer. Semin Cancer Biol (2022) 79:44–57. doi: 10.1016/j.semcancer.2020.06.016 32623044

[17] Rysman E Brusselmans K Scheys K Timmermans L Derua R Munck S. *De Novo* Lipogenesis Protects Cancer Cells From Free Radicals and Chemotherapeutics by Promoting Membrane Lipid Saturation. Cancer Res (2010) 70(20):8117–26. doi: 10.1158/0008-5472.CAN-09-3871 20876798

[18] Peetla C Bhave R Vijayaraghavalu S Stine A Kooijman E Labhasetwar V. Drug Resistance in Breast Cancer Cells: Biophysical Characterization of and Doxorubicin Interactions With Membrane Lipids. Mol Pharm (2010) 7(6):2334–48. doi: 10.1021/mp100308n PMC2997943 20958074

[19] Wu X Dong Z Wang CJ Barlow LJ Fako V Serrano MA. FASN Regulates Cellular Response to Genotoxic Treatments by Increasing PARP-1 Expression and DNA Repair Activity *via* NF-kappaB and SP1. Proc Natl Acad Sci USA (2016) 113(45):E6965–E73. doi: 10.1073/pnas.1609934113 PMC5111708 27791122

[20] Papaevangelou E Almeida GS Box C deSouza NM Chung YL. The Effect of FASN Inhibition on the Growth and Metabolism of a Cisplatin-Resistant Ovarian Carcinoma Model. Int J Cancer (2018) 143(4):992–1002. doi: 10.1002/ijc.31392 29569717 PMC6055739

[21] Wang T Fahrmann JF Lee H Li YJ Tripathi SC Yue C. JAK/STAT3-Regulated Fatty Acid Beta-Oxidation Is Critical for Breast Cancer Stem Cell Self-Renewal and Chemoresistance. Cell Metab (2018) 27(1):136–50 e5. doi: 10.1016/j.cmet.2017.11.001 29249690 PMC5777338

[22] Tan Z Xiao L Tang M Bai F Li J Li L. Targeting CPT1A-Mediated Fatty Acid Oxidation Sensitizes Nasopharyngeal Carcinoma to Radiation Therapy. Theranostics (2018) 8(9):2329–47. doi: 10.7150/thno.21451 PMC5928893 29721083

[23] Dheeraj A Agarwal C Schlaepfer IR Raben D Singh R Agarwal R. A Novel Approach to Target Hypoxic Cancer Cells *via* Combining Beta-Oxidation Inhibitor Etomoxir With Radiation. Hypoxia (Auckl) (2018) 6:23–33. doi: 10.2147/HP.S163115 30175155 PMC6109663

[24] Du T Sikora MJ Levine KM Tasdemir N Riggins RB Wendell SG. Key Regulators of Lipid Metabolism Drive Endocrine Resistance in Invasive Lobular Breast Cancer. Breast Cancer Res (2018) 20(1):106. doi: 10.1186/s13058-018-1041-8 30180878 PMC6124012

[25] Zhang S Chang W Wu H Wang YH Gong YW Zhao YL. Pan-Cancer Analysis of Iron Metabolic Landscape Across the Cancer Genome Atlas. J Cell Physiol (2020) 235(2):1013–24. doi: 10.1002/jcp.29017 31240715

[26] McCarthy DJ Chen Y Smyth GK. Differential Expression Analysis of Multifactor RNA-Seq Experiments With Respect to Biological Variation. Nucleic Acids Res (2012) 40(10):4288–97. doi: 10.1093/nar/gks042 PMC3378882 22287627

[27] Zhang H Meltzer P Davis S. RCircos: An R Package for Circos 2D Track Plots. BMC Bioinform (2013) 14:244. doi: 10.1186/1471-2105-14-244 PMC3765848 23937229

[28] Friedman J Hastie T Tibshirani R. Regularization Paths for Generalized Linear Models *via* Coordinate Descent. J Stat Softw (2010) 33(1):1–22. doi: 10.18637/jss.v033.i01 20808728 PMC2929880

[29] Zhang Z Kattan MW. Drawing Nomograms With R: Applications to Categorical Outcome and Survival Data. Ann Transl Med (2017) 5(10):211. doi: 10.21037/atm.2017.04.01 28603726 PMC5451623

[30] Alba AC Agoritsas T Walsh M Hanna S Iorio A Devereaux PJ. Discrimination and Calibration of Clinical Prediction Models: Users’ Guides to the Medical Literature. JAMA (2017) 318(14):1377–84. doi: 10.1001/jama.2017.12126 29049590

[31] Subramanian A Tamayo P Mootha VK Mukherjee S Ebert BL Gillette MA. Gene Set Enrichment Analysis: A Knowledge-Based Approach for Interpreting Genome-Wide Expression Profiles. Proc Natl Acad Sci USA (2005) 102(43):15545–50. doi: 10.1073/pnas.0506580102 PMC1239896 16199517

[32] Gong Y Ji P Yang YS Xie S Yu TJ Xiao Y. Metabolic-Pathway-Based Subtyping of Triple-Negative Breast Cancer Reveals Potential Therapeutic Targets. Cell Metab (2021) 33(1):51–64.e9. doi: 10.1016/j.cmet.2020.10.012 33181091

[33] Newman AM Liu CL Green MR Gentles AJ Feng W Xu Y. Robust Enumeration of Cell Subsets From Tissue Expression Profiles. Nat Methods (2015) 12(5):453–7. doi: 10.1038/nmeth.3337 PMC4739640 25822800

[34] Chaudhry A Samstein RM Treuting P Liang Y Pils MC Heinrich JM. Interleukin-10 Signaling in Regulatory T Cells Is Required for Suppression of Th17 Cell-Mediated Inflammation. Immunity (2011) 34(4):566–78. doi: 10.1016/j.immuni.2011.03.018 PMC3088485 21511185

[35] Fontenot JD Gavin MA Rudensky AY. Foxp3 Programs the Development and Function of CD4+CD25+ Regulatory T Cells. Nat Immunol (2003) 4(4):330–6. doi: 10.1038/ni904 12612578

[36] Dirat B Bochet L Dabek M Daviaud D Dauvillier S Majed B. Cancer-Associated Adipocytes Exhibit an Activated Phenotype and Contribute to Breast Cancer Invasion. Cancer Res (2011) 71(7):2455–65. doi: 10.1158/0008-5472.CAN-10-3323 21459803

[37] Sun K Tang S Hou Y Xi L Chen Y Yin J. Oxidized ATM-Mediated Glycolysis Enhancement in Breast Cancer-Associated Fibroblasts Contributes to Tumor Invasion Through Lactate as Metabolic Coupling. EBioMedicine (2019) 41:370–83. doi: 10.1016/j.ebiom.2019.02.025 PMC6442874 30799198

[38] Corn KC Windham MA Rafat M. Lipids in the Tumor Microenvironment: From Cancer Progression to Treatment. Prog Lipid Res (2020) 80:101055. doi: 10.1016/j.plipres.2020.101055 32791170 PMC7674189

[39] Currie E Schulze A Zechner R Walther TC Farese RV Jr. Cellular Fatty Acid Metabolism and Cancer. Cell Metab (2013) 18(2):153–61. doi: 10.1016/j.cmet.2013.05.017 PMC3742569 23791484

[40] Volden PA Skor MN Johnson MB Singh P Patel FN McClintock MK. Mammary Adipose Tissue-Derived Lysophospholipids Promote Estrogen Receptor-Negative Mammary Epithelial Cell Proliferation. Cancer Prev Res (Phila) (2016) 9(5):367–78. doi: 10.1158/1940-6207.CAPR-15-0107 PMC4854771 26862086

[41] Zheng S Zou Y Liang JY Xiao W Yang A Meng T. Identification and Validation of a Combined Hypoxia and Immune Index for Triple-Negative Breast Cancer. Mol Oncol (2020) 14(11):2814–33. doi: 10.1002/1878-0261.12747 PMC7607163 32521117

[42] Zheng S Zou Y Xie X Liang JY Yang A Yu K. Development and Validation of a Stromal Immune Phenotype Classifier for Predicting Immune Activity and Prognosis in Triple-Negative Breast Cancer. Int J Cancer (2020) 147(2):542–53. doi: 10.1002/ijc.33009 32285442

[43] Xie J Zou Y Ye F Zhao W Xie X Ou X. A Novel Platelet-Related Gene Signature for Predicting the Prognosis of Triple-Negative Breast Cancer. Front Cell Dev Biol (2021) 9:795600. doi: 10.3389/fcell.2021.795600 35096824 PMC8790231

[44] Yang D Li Y Xing L Tan Y Sun J Zeng B. Utilization of Adipocyte-Derived Lipids and Enhanced Intracellular Trafficking of Fatty Acids Contribute to Breast Cancer Progression. Cell Commun Signal (2018) 16(1):32. doi: 10.1186/s12964-018-0221-6 29914512 PMC6006729

[45] Wang YY Attane C Milhas D Dirat B Dauvillier S Guerard A. Mammary Adipocytes Stimulate Breast Cancer Invasion Through Metabolic Remodeling of Tumor Cells. JCI Insight (2017) 2(4):e87489. doi: 10.1172/jci.insight.87489 28239646 PMC5313068

[46] Han S Wei R Zhang X Jiang N Fan M Huang JH. CPT1A/2-Mediated FAO Enhancement-A Metabolic Target in Radioresistant Breast Cancer. Front Oncol (2019) 9:1201. doi: 10.3389/fonc.2019.01201 31803610 PMC6873486

[47] Theodoropoulos PC Gonzales SS Winterton SE Rodriguez-Navas C McKnight JS Morlock LK. Discovery of Tumor-Specific Irreversible Inhibitors of Stearoyl CoA Desaturase. Nat Chem Biol (2016) 12(4):218–25. doi: 10.1038/nchembio.2016 PMC4798879 26829472

[48] Alnabulsi A Swan R Cash B Alnabulsi A Murray GI. The Differential Expression of Omega-3 and Omega-6 Fatty Acid Metabolising Enzymes in Colorectal Cancer and Its Prognostic Significance. Br J Cancer (2017) 116(12):1612–20. doi: 10.1038/bjc.2017.135 PMC5518862 28557975

[49] Lynch JT Polanska UM Delpuech O Hancox U Trinidad AG Michopoulos F. Inhibiting PI3Kbeta With AZD8186 Regulates Key Metabolic Pathways in PTEN-Null Tumors. Clin Cancer Res (2017) 23(24):7584–95. doi: 10.1158/1078-0432.CCR-17-0676 28972046

[50] Hilvo M Denkert C Lehtinen L Muller B Brockmoller S Seppanen-Laakso T. Novel Theranostic Opportunities Offered by Characterization of Altered Membrane Lipid Metabolism in Breast Cancer Progression. Cancer Res (2011) 71(9):3236–45. doi: 10.1158/0008-5472.CAN-10-3894 21415164

[51] Jahn SC Solayman MH Lorenzo RJ Langaee T Stacpoole PW James MO. GSTZ1 Expression and Chloride Concentrations Modulate Sensitivity of Cancer Cells to Dichloroacetate. Biochim Biophys Acta (2016) 1860(6):1202–10. doi: 10.1016/j.bbagen.2016.01.024 PMC4837035 26850694

[52] Olesch C Sirait-Fischer E Berkefeld M Fink AF Susen RM Ritter B. S1PR4 Ablation Reduces Tumor Growth and Improves Chemotherapy *via* CD8+ T Cell Expansion. J Clin Invest (2020) 130(10):5461–76. doi: 10.1172/JCI136928 PMC7524469 32663191

[53] Porstmann T Santos CR Griffiths B Cully M Wu M Leevers S. SREBP Activity Is Regulated by Mtorc1 and Contributes to Akt-Dependent Cell Growth. Cell Metab (2008) 8(3):224–36. doi: 10.1016/j.cmet.2008.07.007 PMC2593919 18762023

[54] Ricoult SJ Yecies JL Ben-Sahra I Manning BD. Oncogenic PI3K and K-Ras Stimulate *De Novo* Lipid Synthesis Through Mtorc1 and SREBP. Oncogene (2016) 35(10):1250–60. doi: 10.1038/onc.2015.179 PMC4666838 26028026

[55] Freeman GJ Long AJ Iwai Y Bourque K Chernova T Nishimura H. Engagement of the PD-1 Immunoinhibitory Receptor by a Novel B7 Family Member Leads to Negative Regulation of Lymphocyte Activation. J Exp Med (2000) 192(7):1027–34. doi: 10.1084/jem.192.7.1027 PMC2193311 11015443

[56] Yaqoob P Newsholme EA Calder PC. Inhibition of Natural Killer Cell Activity by Dietary Lipids. Immunol Lett (1994) 41(2-3):241–7. doi: 10.1016/0165-2478(94)90140-6 8002045

[57] Herber DL Cao W Nefedova Y Novitskiy SV Nagaraj S Tyurin VA. Lipid Accumulation and Dendritic Cell Dysfunction in Cancer. Nat Med (2010) 16(8):880–6. doi: 10.1038/nm.2172 PMC2917488 20622859

[58] Veglia F Tyurin VA Mohammadyani D Blasi M Duperret EK Donthireddy L. Lipid Bodies Containing Oxidatively Truncated Lipids Block Antigen Cross-Presentation by Dendritic Cells in Cancer. Nat Commun (2017) 8(1):2122. doi: 10.1038/s41467-017-02186-9 29242535 PMC5730553

[59] Ali HR Chlon L Pharoah PD Markowetz F Caldas C. Patterns of Immune Infiltration in Breast Cancer and Their Clinical Implications: A Gene-Expression-Based Retrospective Study. PloS Med (2016) 13(12):e1002194. doi: 10.1371/journal.pmed.1002194 27959923 PMC5154505

[60] Bense RD Sotiriou C Piccart-Gebhart MJ Haanen J van Vugt M de Vries EGE. Relevance of Tumor-Infiltrating Immune Cell Composition and Functionality for Disease Outcome in Breast Cancer. J Natl Cancer Inst (2017) 109(1). doi: 10.1093/jnci/djw192 PMC6284248 27737921

[61] Freemerman AJ Johnson AR Sacks GN Milner JJ Kirk EL Troester MA. Metabolic Reprogramming of Macrophages: Glucose Transporter 1 (GLUT1)-Mediated Glucose Metabolism Drives a Proinflammatory Phenotype. J Biol Chem (2014) 289(11):7884–96. doi: 10.1074/jbc.M113.522037 PMC3953299 24492615

[62] Howie D Cobbold SP Adams E Ten Bokum A Necula AS Zhang W. Foxp3 Drives Oxidative Phosphorylation and Protection From Lipotoxicity. JCI Insight (2017) 2(3):e89160. doi: 10.1172/jci.insight.89160 28194435 PMC5291728

[63] Di Giacinto C Marinaro M Sanchez M Strober W Boirivant M. Probiotics Ameliorate Recurrent Th1-Mediated Murine Colitis by Inducing IL-10 and IL-10-Dependent TGF-Beta-Bearing Regulatory Cells. J Immunol (2005) 174(6):3237–46. doi: 10.4049/jimmunol.174.6.3237 15749854

[64] Fujisaki K Fujimoto H Sangai T Nagashima T Sakakibara M Shiina N. Cancer-Mediated Adipose Reversion Promotes Cancer Cell Migration *via* IL-6 and MCP-1. Breast Cancer Res Treat (2015) 150(2):255–63. doi: 10.1007/s10549-015-3318-2 25721605

[65] Lopes-Coelho F Andre S Felix A Serpa J. Breast Cancer Metabolic Cross-Talk: Fibroblasts are Hubs and Breast Cancer Cells Are Gatherers of Lipids. Mol Cell Endocrinol (2018) 462(Pt B):93–106. doi: 10.1016/j.mce.2017.01.031 28119133

[66] Santi A Caselli A Ranaldi F Paoli P Mugnaioni C Michelucci E. Cancer Associated Fibroblasts Transfer Lipids and Proteins to Cancer Cells Through Cargo Vesicles Supporting Tumor Growth. Biochim Biophys Acta (2015) 1853(12):3211–23. doi: 10.1016/j.bbamcr.2015.09.013 26384873

[67] Hultsch S Kankainen M Paavolainen L Kovanen RM Ikonen E Kangaspeska S. Association of Tamoxifen Resistance and Lipid Reprogramming in Breast Cancer. BMC Cancer (2018) 18(1):850. doi: 10.1186/s12885-018-4757-z 30143015 PMC6109356

[68] Nguyen VT Barozzi I Faronato M Lombardo Y Steel JH Patel N. Differential Epigenetic Reprogramming in Response to Specific Endocrine Therapies Promotes Cholesterol Biosynthesis and Cellular Invasion. Nat Commun (2015) 6:10044. doi: 10.1038/ncomms10044 26610607 PMC4674692

