# Supplementary material for: Prognosis and Dissection of Immunosuppressive Microenvironment in Breast Cancer Based on Fatty Acid Metabolism-Related Signature
Source: Front Immunol. 2022 Mar 31;13:843515. doi: 10.3389/fimmu.2022.843515 (PMC9009264; doi:10.3389/fimmu.2022.843515)
Supplement: Supplementary file 1 [file DataSheet_1.docx]

Supplementary Material

**SUPPLEMENTARY TABLE**

**Supplementary Table S1**. Primers sequences were shown.

| **Supplementary Table S1. Primers for qRT-PCR detection** | | |
| --- | --- | --- |
| ELOVL1 | Forward | AACTCTTCCGTGCATGTCATAAT |
|  | Reverse | TGCTTTTTCCACCAAAGGTAGG |
| LTA4H | Forward | AGACAAAGTTACAAGGGATCGC |
|  | Reverse | AGATATGGGTGTTCCTTCCCAG |
| β-actin | Forward | CATGTACGTTGCTATCCAGGC |
|  | Reverse | CTCCTTAATGTCACGCACGAT |
