# Supplementary material for: Prognosis and Dissection of Immunosuppressive Microenvironment in Breast Cancer Based on Fatty Acid Metabolism-Related Signature
Source: Front Immunol. 2022 Mar 31;13:843515. doi: 10.3389/fimmu.2022.843515 (PMC9009264; doi:10.3389/fimmu.2022.843515)

Supplementary Material

**SUPPLEMENTARY FIGURE LEGEND**

**Supplementary Figure 1**. Identification of 269 reliable fatty acid metabolism-related genes. (A) Venn diagram to identify 269 overlapping FMGs in TCGA-BRCA, METABRIC and GSE96058 datasets. (B) The information of 269 eligible FMGs.

**Supplementary Figure 2**. Expression level of ELOVL1 and LTA4H in human breast cancer cell lines. (A) Expression level of ELOVL1. (B) Expression level of LTA4H.

**Supplementary Figure 3**. Expression level of immune checkpoints in two validation sets. (A) METABRIC dataset. (B) GSE96058 dataset.

**Supplementary Figure 4**. Landscape of TME between high- and low-FMI groups in Basal subtype or non-Basal subtype. Violin plots of the proportions of 22 immune infiltrating cells in Basal subtype (A) and non-Basal subtype.


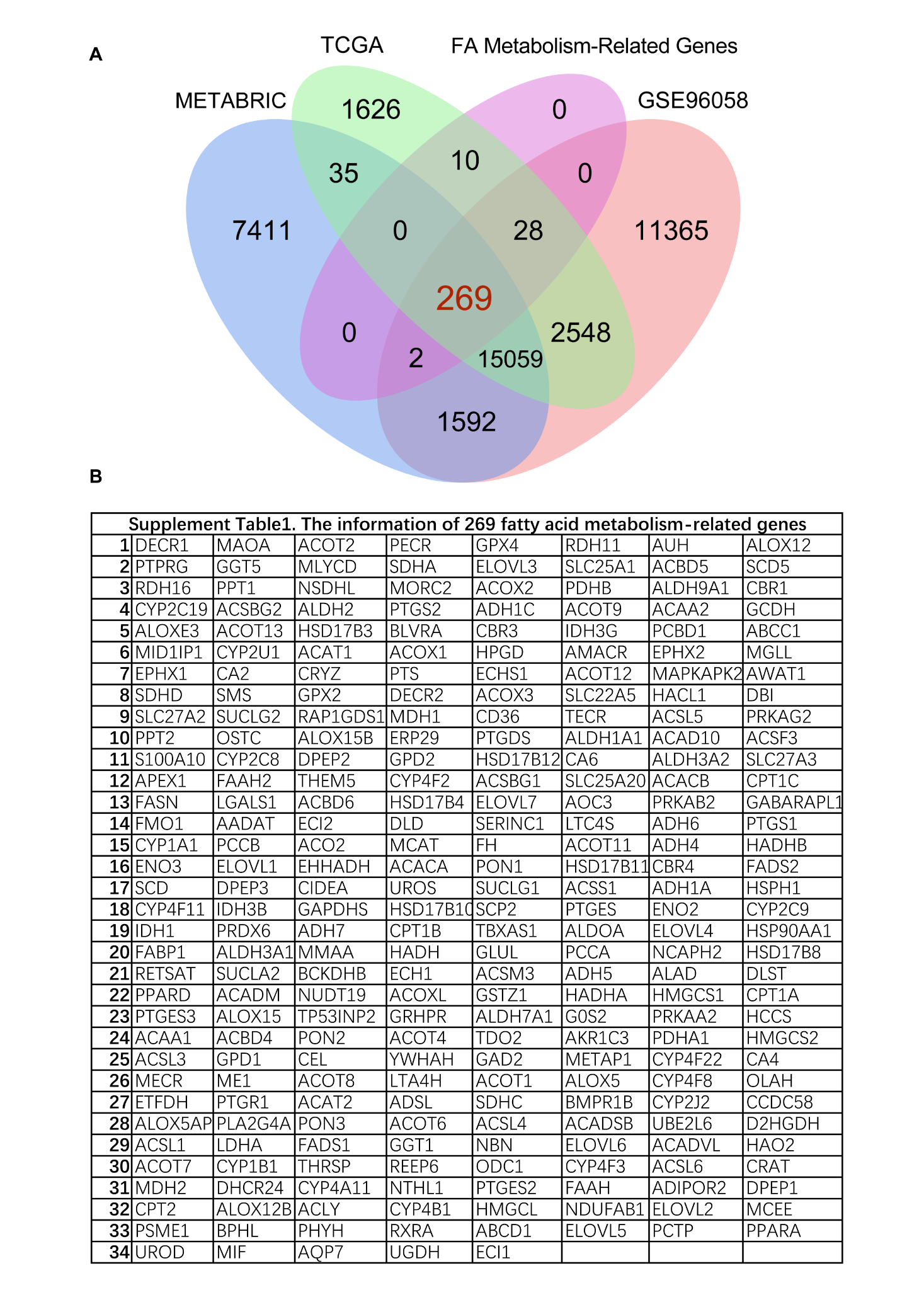


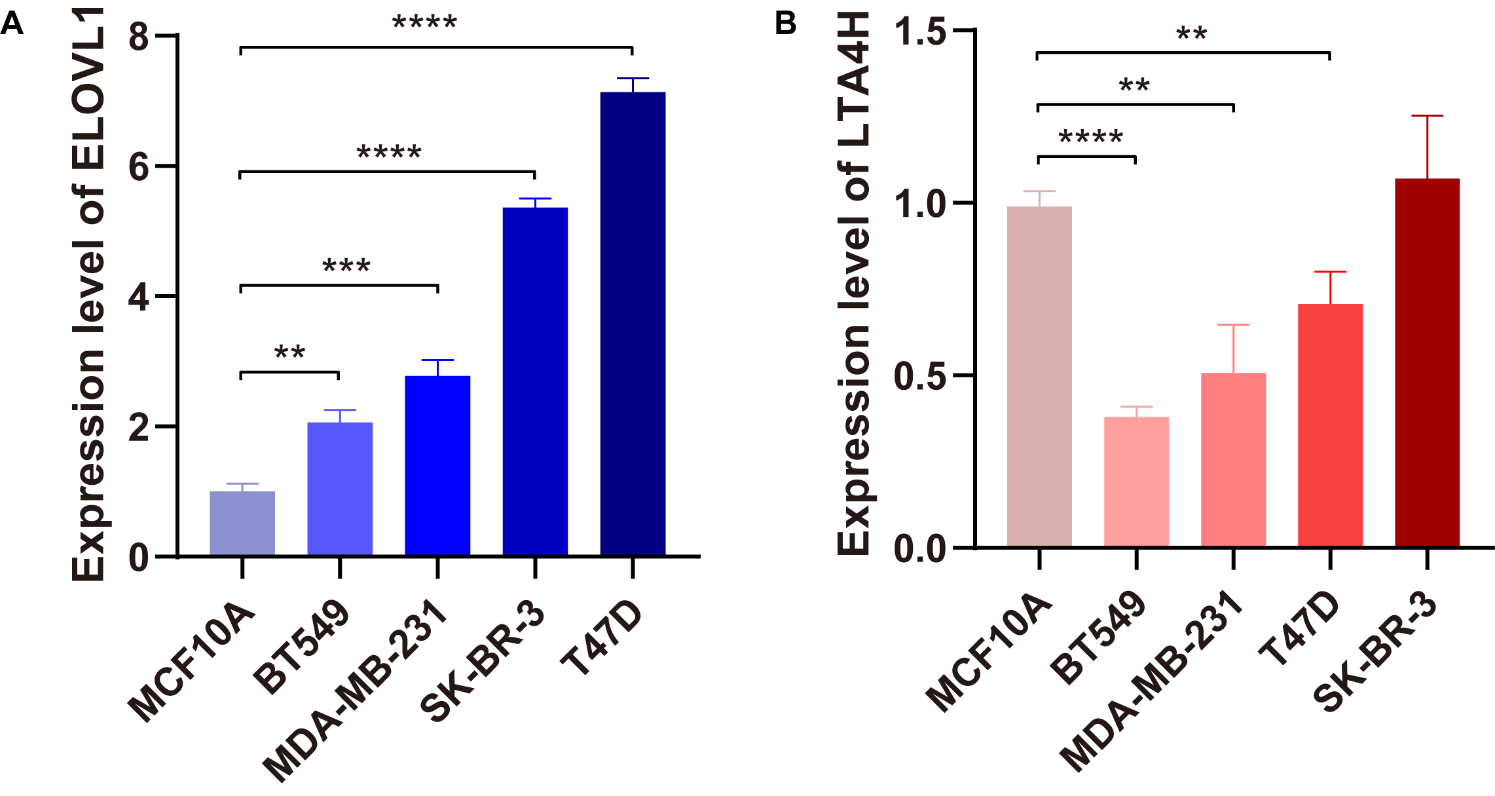


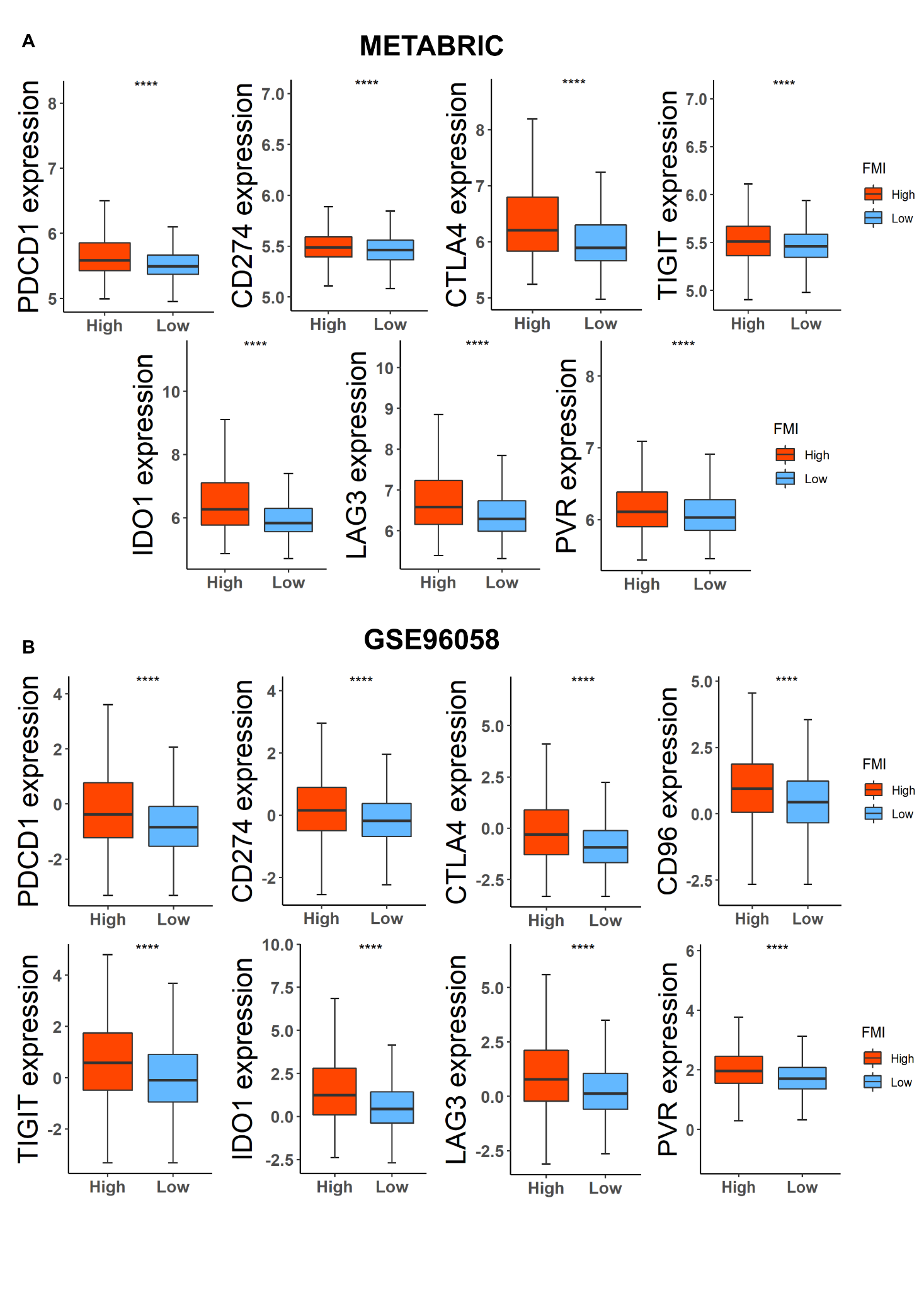

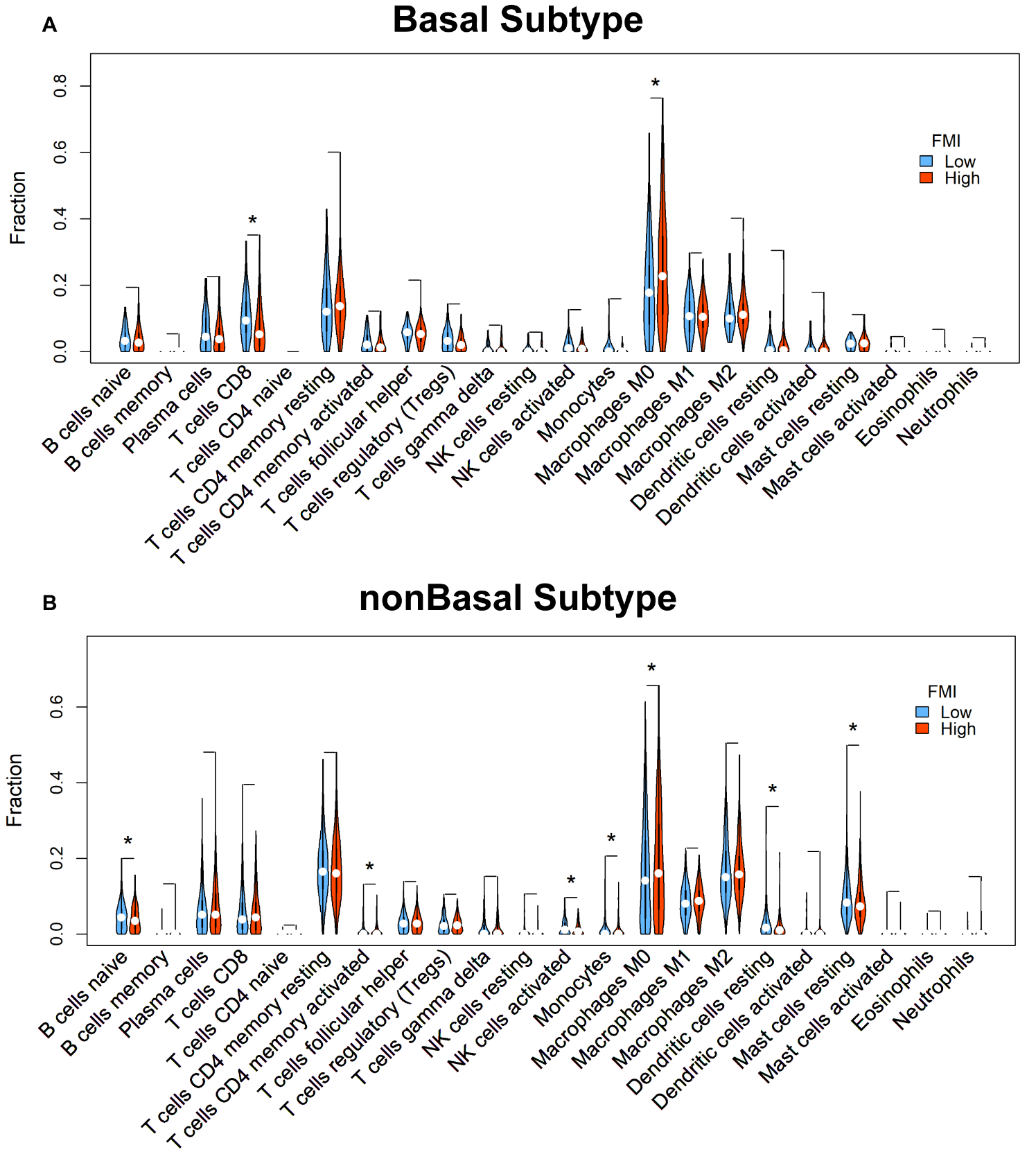

Supplement: Supplementary file 2 [file DataSheet_2.docx]
